# Clinical Profile and Short-Term Outcomes of Patients With Intradialytic Hypertension Undergoing Maintenance Hemodialysis in a Tertiary Care Centre: A Prospective Cohort Study

**DOI:** 10.7759/cureus.104398

**Published:** 2026-02-27

**Authors:** Mithran B Raja, Karthikeyan G, Sivasankari G, Haridoss Sripriya Vasudevan, Yogesh Subramanian

**Affiliations:** 1 Institute of Internal Medicine, Madras Medical College and Rajiv Gandhi Government General Hospital, Chennai, IND; 2 Business Administration, Birla Institute of Technology And Science, Pilani, Pilani, IND; 3 Health Profession Education, Sri Balaji Vidyapeeth University, Pillaiyarkuppam, IND

**Keywords:** cardiovascular adverse events, chronic kidney disease (ckd), dialysis related complications, intradialytic hypertension, maintenance hemodialysis, morbidity and mortality

## Abstract

Introduction

Intradialytic hypertension (IDH) is an underrecognized complication during hemodialysis that has been associated with major adverse cardiovascular events (MACE) and mortality. Prospective studies from tertiary centers in developing countries are limited. The present study was carried out to assess the incidence of IDH and its association with demographic and clinical characteristics and short-term outcomes among patients receiving maintenance hemodialysis.

Materials and methods

In this prospective cohort study, 103 adult patients receiving maintenance hemodialysis were followed for six months at Madras Medical College and Rajiv Gandhi Government General Hospital, a tertiary care institution in South India. Blood pressure data were taken before, during, and after dialysis sessions. IDH was defined as a ≥10 mmHg rise in systolic blood pressure between pre- and post-dialysis in at least two consecutive sessions. Clinical, biochemical, and dialysis-related characteristics were compared between the IDH and non-IDH groups. Intradialytic adverse events, hospitalization, cardiovascular events, and mortality were the outcomes reported.

Results

The incidence of IDH was 27.2%. IDH patients were older, had higher BMI, had longer dialysis history (all p < 0.01), and were more likely to have diabetes, coronary artery disease, and a previous stroke. They had lower hemoglobin, calcium levels, and greater sodium and phosphorus levels (p < 0.01). Dialysate sodium, ultrafiltration volume and rate, interdialytic weight increase, and dialysate temperature were all significantly higher in the IDH group (p < 0.001). IDH was associated with increased intradialytic adverse events (55.6% vs 21.2%), hospitalization (60.0% vs 19.3%), cardiovascular events (58.8% vs 20.9%), and mortality (66.7% vs 23.9%) (all p < 0.05). The survival analysis revealed significantly reduced cardiovascular event-free, hospitalization-free, and overall survival.

Conclusion

IDH affected more than one-quarter of patients and was significantly associated with adverse short-term outcomes in patients undergoing maintenance hemodialysis.

## Introduction

Chronic kidney disease (CKD) has become a major global health burden, affecting roughly 10% of the population, with many patients advancing to end-stage renal disease (ESRD), which requires continuous hemodialysis [1]. Although hemodialysis is a life-saving therapy, it is commonly accompanied by cardiovascular and hemodynamic abnormalities, which contribute significantly to morbidity and mortality in this population [2,3].

Blood pressure (BP) instability during hemodialysis is common, and it can present as either intradialytic hypotension or intradialytic hypertension (IDH). IDH is distinguished by a paradoxical rise in systolic blood pressure during or immediately following dialysis, and is most generally described as an increase of more than 10 mmHg from pre- to post-dialysis over multiple sessions [4,5]. IDH, in comparison to intradialytic hypotension, is generally underappreciated, despite mounting evidence of its negative clinical ramifications.

The pathophysiology of IDH is complex and poorly understood. Proposed explanations include prolonged volume overload, increased systemic vascular resistance, endothelial dysfunction, neurohormonal activation, sodium retention during dialysis, and variations in dialysate composition and ultrafiltration rate [6-8].

The reported prevalence of IDH ranges from 5% to more than 40%, owing to variation in criteria and study populations [4,9]. A recent systematic review and meta-analysis found that the pooled prevalence was 26.6%, with a corresponding increase in mortality risk [10]. Recurrent IDH has also been linked to an increased risk of hospitalization and cardiovascular events [11]. However, there is a dearth of prospective data from tertiary care centers in developing countries. Hence, this study was carried out to assess the incidence of IDH and its association with demographic and clinical characteristics and short-term outcomes among patients receiving maintenance hemodialysis.

## Materials and methods

This prospective cohort study was conducted over a period of six months in the maintenance hemodialysis unit of Madras Medical College and Rajiv Gandhi Government General Hospital, a tertiary care teaching hospital in Chennai, India. The study was approved by theInstitutional Ethics Committee, Madras Medical College (approval number: IEC/MMC/Approval/39122025), and signed informed consent was taken from all patients. The Declaration of Helsinki (2013) was followed throughout the investigation [12].

Eligibility criteria

Adult patients (≥18 years) with ESRD receiving maintenance hemodialysis for at least two months were enrolled using a consecutive sampling technique. All patients undergoing hemodialysis at least twice a week, taking at least one antihypertensive medication, and providing written informed consent were included in the study. Patients with hemodynamic instability, active sepsis (expected survival <48 hours), poor dialysis compliance (missed ≥2 sessions per month), or severe comorbid conditions such as active malignancy, morbid obesity (BMI ≥40 kg/m²), severe malnutrition, or terminal illness were excluded from the study.

Sample size

The sample size was calculated using an IDH prevalence of 21.9% [11], a two-sided alpha of 0.05, and an absolute precision of 8%, yielding a required sample of approximately 103 participants.

Intervention

All patients underwent hemodialysis three times per week using conventional bicarbonate dialysate. Dialysis recommendations, which included duration, blood and dialysate flow rates, dialysate sodium and calcium concentrations, dialysate temperature, and ultrafiltration (UF) objectives, were customized to clinical requirements.

Data collection

Blood pressure was recorded with calibrated automated instruments and appropriate cuff sizes from the non-access upper arm in the supine position. Measurements were taken in the supine position after at least five minutes of rest. Blood pressure was measured before dialysis initiation, hourly during dialysis, and 10 minutes after the conclusion of dialysis. IDH was defined as a systolic blood pressure increase of ≥10 mmHg between pre- and post-dialysis in at least two consecutive dialysis sessions [4,5].

Baseline data included demographic characteristics, comorbidities, dialysis history, and antihypertensive drug use. Dialysis parameters recorded were session duration, blood and dialysate flow rates, dialysate sodium, and temperature; prescribed and achieved UF volumes; UF rate (mL/kg/h); and heparin use. Intradialytic interventions done for those with IDH included IV fluids or antihypertensives (e.g., labetalol), UF interruption, and saline bolus.

Outcomes

The primary outcome was the incidence of IDH. Secondary outcomes included intradialytic adverse events, hospitalization, cardiovascular events, bone mineral disease, and mortality during follow-up. Major adverse cardiovascular events (MACE) were defined as a composite outcome comprising all-cause mortality, myocardial infarction, stroke, hospitalization for heart failure, or cardiovascular revascularization procedures.

Data analysis

The data were analyzed with IBM SPSS Statistics for Windows, version 26.0 (IBM Corp., Armonk, New York, United States). Continuous variables were presented as mean ± standard deviation (SD), whereas categorical variables were represented as frequencies and percentages. Comparisons between groups were carried out using the chi-square test or Fisher's exact test for categorical variables and independent t-tests for continuous variables. Kaplan Meier survival studies were performed to assess hospitalization, cardiovascular events, and mortality rates. A two-tailed p-value <0.05 indicated statistical significance.

## Results

A total of 103 patients undergoing maintenance hemodialysis were assessed in this prospective observational study. IDH was identified in 28 patients (27.2%), whereas 75 patients (72.8%) did not develop IDH, indicating that more than one-quarter of the study population encountered this consequence. The mean age was 55.7 ± 10.7 years, and 68 (66.0%) were male. The population had a significant cardiometabolic burden, with 56 (54.4%) having diabetes, 31 (30.1%) having coronary artery disease, 27 (26.2%) having heart failure, and 11 (10.7%) having experienced a previous stroke. The average body mass index was 25.8 ± 4.1 kg/m², and the dialysis duration was 3.4 ± 2.0 years. Dialyzable antihypertensive medicines were prescribed to 49 patients (47.6%) (Table 1).

**Table 1:** Baseline demographic and clinical characteristics of the study population (N=103) Values are given in n (%) and mean ± SD SD: Standard deviation

Variable	Category	Value
Age (years), mean ± SD	—	55.7 ± 10.7
Sex, n (5)	Male	68 (66.0)
	Female	35 (34.0)
Body mass index (kg/m²), mean ± SD	—	25.8 ± 4.1
Dialysis duration (years), mean ± SD	—	3.4 ± 2.0
Smoking history, n (%)	Yes	32 (31.1)
	No	71 (68.9)
Alcohol use, n (%)	Yes	28 (27.2)
	No	75 (72.8)
Dialyzable antihypertensive use, n (%)	Yes	49 (47.6)
	No	54 (52.4)
Diabetes mellitus, n (%)	Yes	56 (54.4)
	No	47 (45.6)
Coronary artery disease, n (%)	Yes	31 (30.1)
	No	72 (69.9)
Heart failure, n (%)	Yes	27 (26.2)
	No	76 (73.8)
Stroke, n (%)	Yes	11 (10.7)
	No	92 (89.3)

Patients with IDH demonstrated a distinct high-risk phenotype. Compared to non-IDH patients, IDH patients were considerably older (59.3 ± 10.2 vs 51.7 ± 11.4 years), had a higher BMI (28.4 ± 4.1 vs 24.6 ± 3.7 kg/m²), and had a longer dialysis history (4.5 ± 2.3 vs 3.0 ± 1.8 years) (p < 0.01). IDH was found in 35.7% of diabetics against 17.0% of non-diabetics, 45.2% of patients with coronary artery disease versus 19.4% without, and 63.6% of patients with a previous stroke versus 25.6% without. Dialyzable antihypertensive medications were associated with higher rates of IDH (40.8% vs 21.6%) (all p < 0.05) (Table 2).

**Table 2:** Association of IDH with baseline demographic and clinical characteristics of the study population (N=103) Data presented as n (%) and mean ± SD; *Chisquare test; **Fischer's exact test; ^$^Independent t-test; P-value <0.05 is significant IDH: intradialytic hypertension; SD: standard deviation

Variable	Category	IDH Present, n (%)/mean ± SD	IDH Absent, n (%)/mean ± SD	Test statistics, χ2/t value	p value
Age (years)	—	59.3 ± 10.2	51.7 ± 11.4	t= 3.09^$^	0.002
Sex	Male	18 (26.5)	50 (73.5)	χ2= 0.05^*^	0.82
	Female	10 (28.6)	25 (71.4)		
Body mass index (kg/m²)	—	28.4 ± 4.1	24.6 ± 3.7	t= 4.50^$^	<0.001
Dialysis duration (years)	—	4.5 ± 2.3	3.0 ± 1.8	t= 3.48^$^	<0.001
Smoking history	Yes	12 (37.5)	20 (62.5)	χ2= 2.49^*^	0.11
	No	16 (21.3)	55 (78.7)		
Alcohol use	Yes	10 (35.7)	18 (64.3)	χ2= 1.41^*^	0.23
	No	18 (24)	57 (76)		
Dialyzable anti-hypertensive use	Yes	20 (40.8)	29 (59.2)	χ2= 8.77^*^	0.003
	No	8 (21.6)	46 (78.4)		
Diabetes mellitus	Yes	20 (35.7)	36 (64.3)	χ2= 4.51^*^	0.03
	No	8 (17.0)	39 (83.0)		
Coronary artery disease	Yes	14 (45.2)	17 (54.8)	χ2= 7.24^*^	0.007
	No	14 (19.4)	58 (80.6)		
Heart failure	Yes	11 (40.7)	16 (59.3)	χ2= 3.39^*^	0.06
	No	17 (22.4)	59 (77.6)		
Stroke	Yes	7 (63.6)	4 (36.4)	χ2= 6.33^**^	0.01
	No	21 (25.6)	71 (74.4)		

IDH patients had significantly lower hemoglobin levels (9.8 ± 1.2 vs 10.6 ± 1.5 g/dL), serum calcium (8.2 ± 0.6 vs 8.7 ± 0.7 mg/dL), and higher serum sodium (142.2 ± 3.3 vs 139.0 ± 3.6 mEq/L) and phosphorus (5.6 ± 0.9 vs 4.7 ± 0.9 mg/dL) than patients without IDH (all p ≤ 0.01), suggesting a significant association with abnormalities in fluid balance and mineral homeostasis (Table 3).

**Table 3:** Association of biochemical parameters and hemoglobin with IDH (N = 103) ^$^ Independent t-test; P value <0.05 is significant IDH: intradialytic hypertension; SD: standard deviation

Variable	IDH Present (n = 28), mean ± SD	IDH Absent (n = 75), mean ± SD	t value^$^	p value
Hemoglobin (g/dL)	9.8 ± 1.2	10.6 ± 1.5	2.53	0.01
Serum Sodium (mEq/L)	142.2 ± 3.3	139.0 ± 3.6	4.10	<0.001
Serum Potassium (mEq/L)	4.8 ± 0.6	4.7 ± 0.7	0.67	0.5
Serum Calcium (mg/dL)	8.2 ± 0.6	8.7 ± 0.7	3.35	0.001
Serum Phosphorus (mg/dL)	5.6 ± 0.9	4.7 ± 0.9	4.51	<0.001
Intact PTH (pg/mL)	315 ± 68	298 ± 64	1.18	0.24
Alkaline Phosphatase (IU/L)	138 ± 32	131 ± 30	1.03	0.30

Dialysis-related factors had some of the most significant associations with IDH. Patients with IDH had higher dialysate sodium (140.8 ± 2.4 vs 138.2 ± 2.6 mEq/L), higher ultrafiltration volume (3.1 ± 0.7 vs 2.4 ± 0.6 L/session), higher ultrafiltration rate (11.2 ± 2.1 vs 8.9 ± 1.8 mL/kg/h), higher dialysate temperature (36.4 ± 0.4 °C), and greater interdialytic weight gain (3.3 ± 0.8 vs 2.4 ± 0.7 kg) (all p < 0.001) (Table 4).

**Table 4:** Association between dialysis-related factors and IDH (N = 103) ^$^ Independent t-test; P value <0.05 is significant IDH: intradialytic hypertension; SD: standard deviation; UF: ultrafiltration

Variable	IDH Present (n = 28), mean ± SD	IDH Absent (n = 75), mean ± SD	t value^$^	p value
Dialysate Sodium (mEq/L)	140.8 ± 2.4	138.2 ± 2.6	4.60	<0.001
UF Volume (L/session)	3.1 ± 0.7	2.4 ± 0.6	5.03	<0.001
UF Rate (mL/kg/hr)	11.2 ± 2.1	8.9 ± 1.8	5.51	<0.001
Dialysate Temperature (°C)	36.8 ± 0.3	36.4 ± 0.4	4.80	<0.001
Interdialytic Weight Gain (kg)	3.3 ± 0.8	2.4 ± 0.7	5.58	<0.001

Table 5 shows that patients with IDH have higher pre-dialysis systolic BP (166.8 ± 15.6 vs 159.4 ± 15.2 mmHg) than patients with IDH (p = 0.03).

**Table 5:** Association between BP parameters and IDH (N = 103) ^$^ Independent t-test; P value <0.05 is significant IDH: intradialytic hypertension; SD: standard deviation; BP: blood pressure

Variable	IDH Present (n = 28), mean ± SD	IDH Absent (n = 75), mean ± SD	t value^$^	p value
Pre-dialysis Systolic BP (mmHg)	166.8 ± 15.6	159.4 ± 15.2	2.18	0.03
Pre-dialysis Diastolic BP (mmHg)	89.6 ± 9.8	86.9 ± 9.5	1.27	0.21
Post-dialysis Systolic BP (mmHg)	172.9 ± 17.8	165.1 ± 18.4	1.93	0.06
Post-dialysis Diastolic BP (mmHg)	92.4 ± 9.6	88.6 ± 9.3	1.83	0.07

Additionally, IDH was linked to significantly poorer clinical results. Table 6 presents the distribution of IDH status across short-term clinical outcomes. Among patients who experienced intradialytic adverse events, 55.6% had IDH compared with 44.4% without IDH. A similar distribution was observed for major clinical outcomes: 60.0% of hospitalized patients had IDH, while 40.0% did not; 58.8% of patients with cardiovascular events had IDH compared with 41.2% without; and 66.7% of patients who died had IDH. All these associations were statistically significant (p < 0.05). In contrast, the distribution of bone mineral disease did not differ significantly according to IDH status (39.1% vs 60.9%, p > 0.05). These findings indicate a significant association between IDH and adverse short-term outcomes in this cohort

**Table 6:** Association between IDH and clinical outcomes among hemodialysis patients (N = 103) *Chi-square/Fisher's exact test; P value <0.05 is significant; Values are presented as row percentages, representing the proportion of patients with and without IDH within each outcome category. IDH: intradialytic hypertension

Variable	Category	IDH Present, n (%)	IDH Absent, n (%)	χ2 value^*^	p-value
Intradialytic Adverse Events	Yes	10 (55.6)	8 (44.4)	7.22	0.007
	No	18 (21.2)	67 (78.8)		
Hospitalization During Follow-up	Yes	12 (60.0)	8 (40.0)	13.50	<0.001
	No	16 (19.3)	67 (80.7)		
Cardiovascular Events	Yes	10 (58.8)	7 (41.2)	8.47	0.003
	No	18 (20.9)	68 (79.1)		
Bone Mineral Disease Progression	Yes	9 (39.1)	14 (60.9)	2.14	0.14
	No	19 (23.8)	61 (76.2)		
Mortality	Yes	6 (66.7)	3 (33.3)	5.73	0.02
	No	22 (23.9)	72 (76.1)		

**Figure 1:**
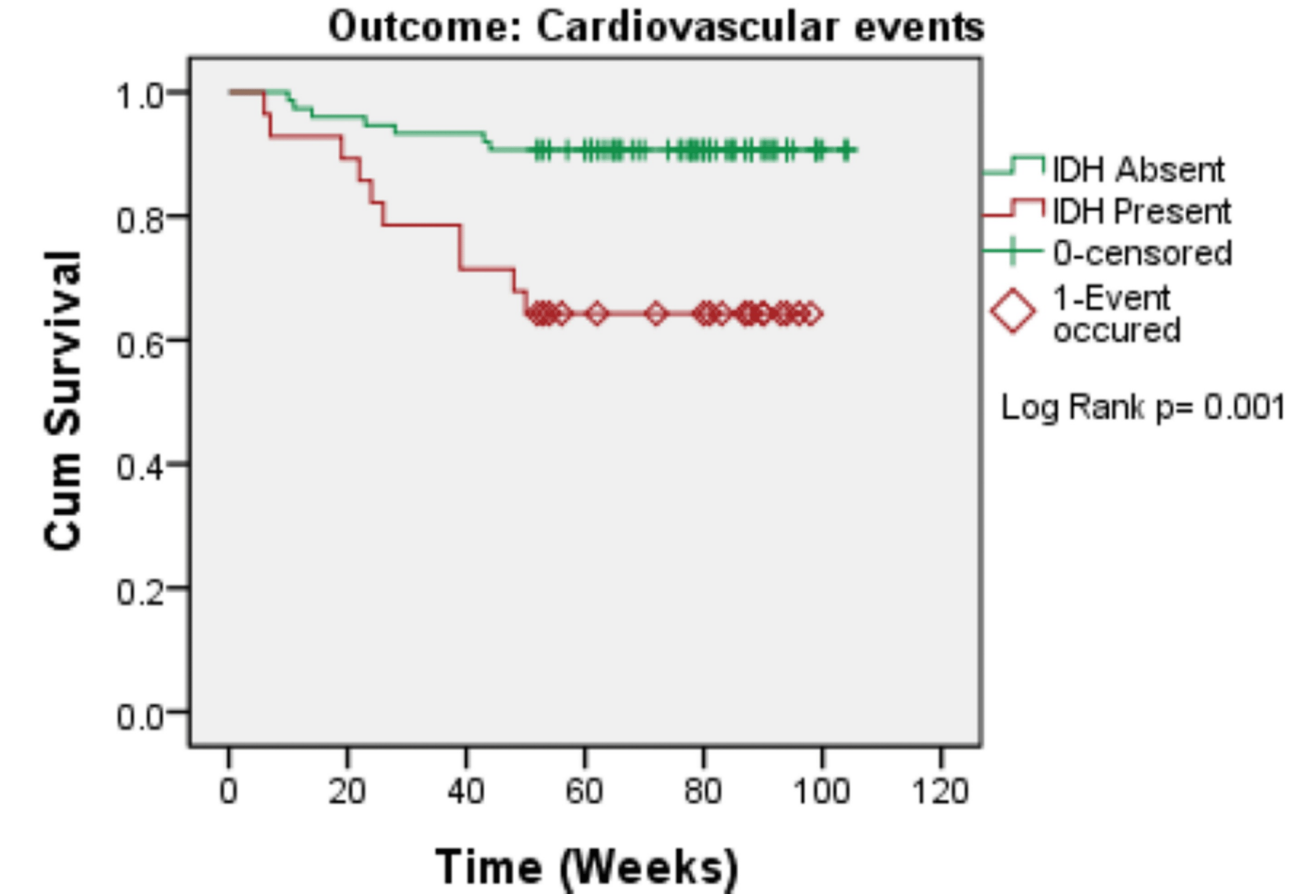
Kaplan–Meier curve for cardiovascular events according to IDH status (N=103) IDH: intradialytic hypertension

**Figure 2:**
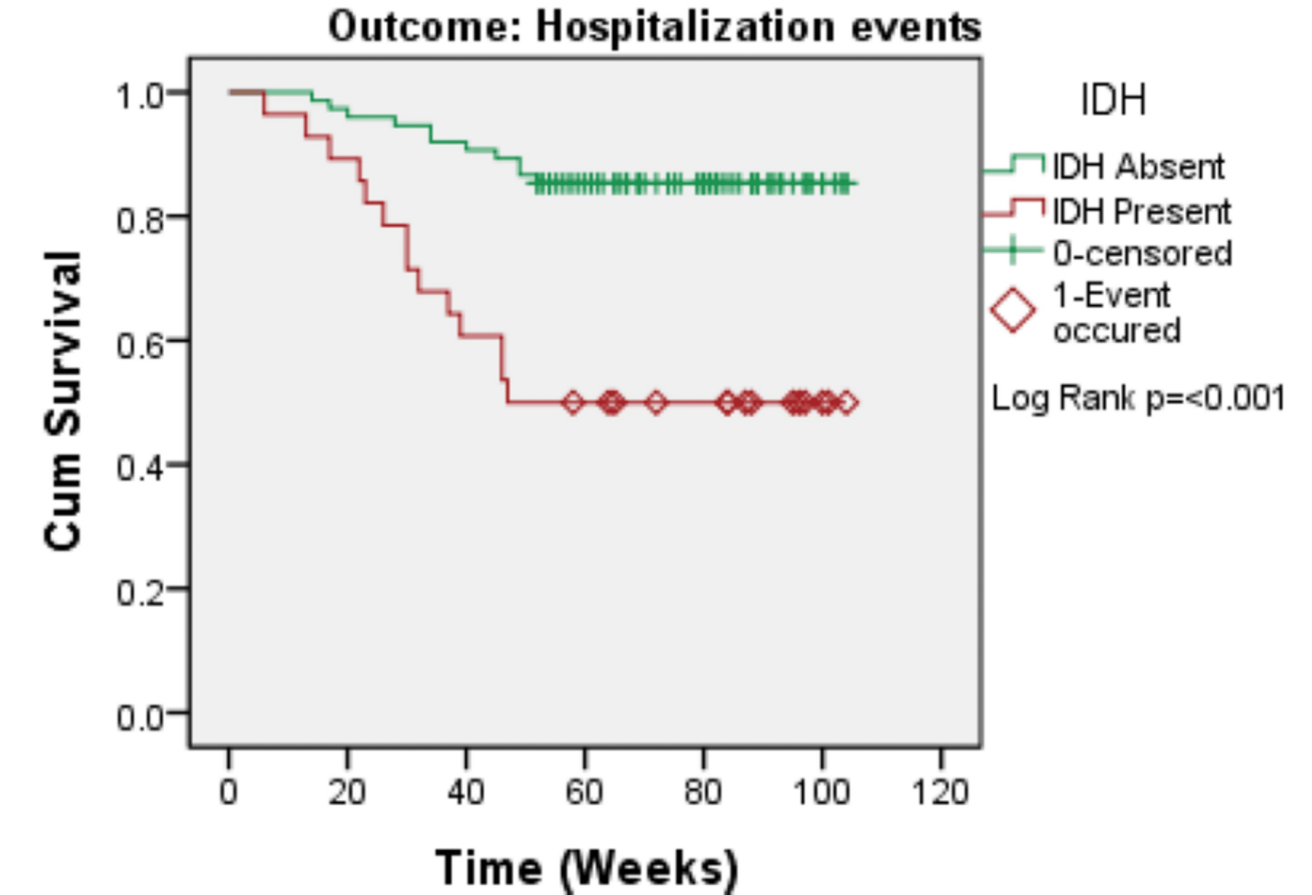
Kaplan–Meier curve for hospitalization events according to IDH status (N=103) IDH: intradialytic hypertension

**Figure 3:**
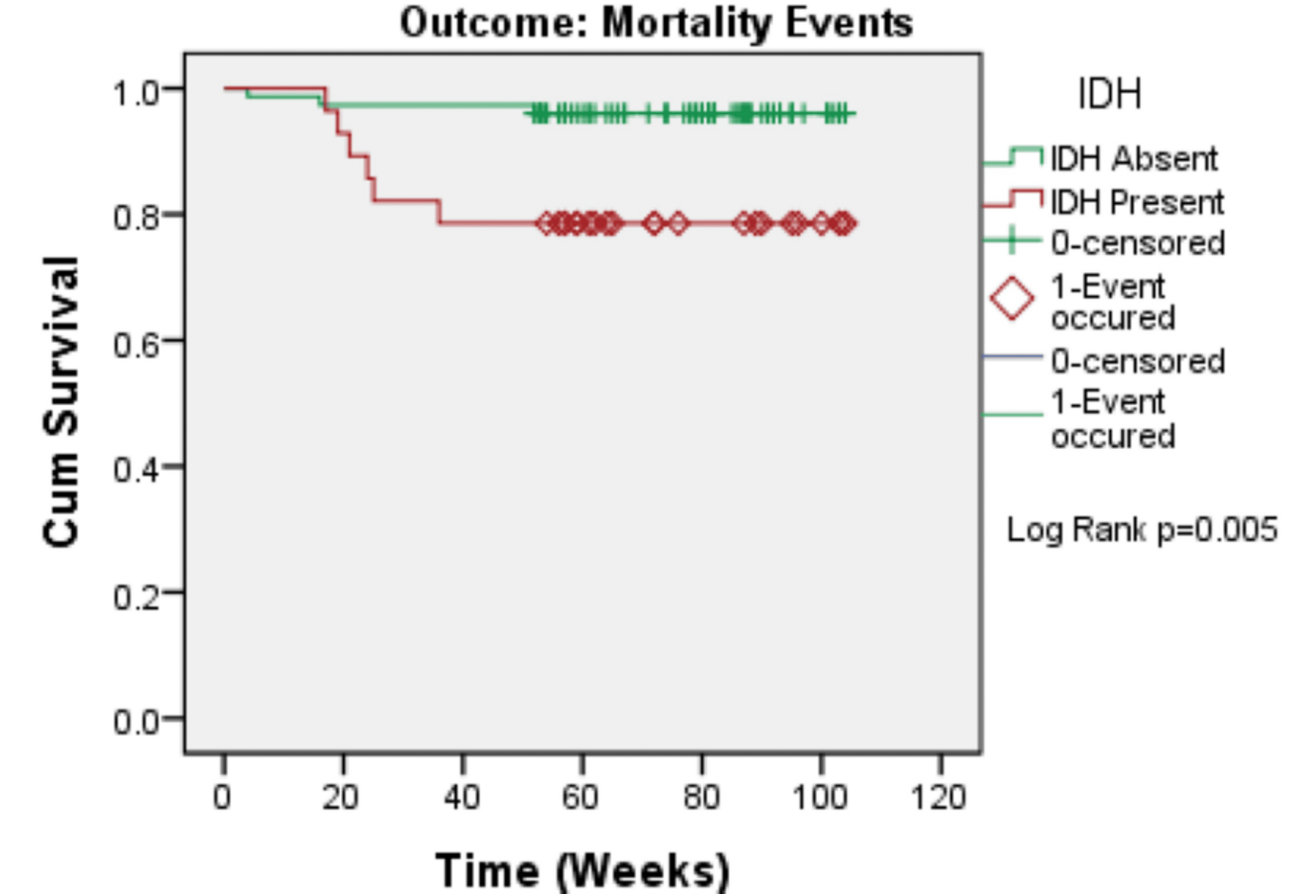
Kaplan–Meier curve for mortality events according to IDH status (N=103) IDH: intradialytic hypertension

The Kaplan-Meier survival analysis revealed that patients with IDH had significantly poorer clinical outcomes than those without IDH. Patients with IDH had significantly lower cardiovascular event-free survival (log-rank p = 0.001), hospitalization-free survival (log-rank p < 0.001), and overall survival (log-rank p = 0.005). In all three outcomes, the survival curves split early and remained divergent during follow-up, demonstrating that IDH has a long-term negative impact on short-term clinical outcomes (Figures 1-3).

IDH affected more than one-quarter of patients and was associated with adverse clinical profiles, unfavorable dialysis characteristics, and higher rates of intradialytic complications, hospitalizations, cardiovascular events, and mortality, suggesting a significant association between IDH and adverse short-term outcomes in patients undergoing maintenance hemodialysis.

## Discussion

IDH is increasingly recognized as a clinically significant blood pressure pattern among patients undergoing maintenance hemodialysis. Multiple studies have demonstrated that IDH is prevalent and strongly associated with adverse outcomes. Kale et al. reported IDH in 21.9% of patients monitored over 12 months [11], while Tariq et al. observed a prevalence of 19.4% [4]. Sherif et al. reported that 34% of patients experienced IDH, occurring in 1.9% of sessions [13], whereas Patrice et al. documented a markedly higher prevalence of 48.3%, with 93.7% of patients experiencing at least one episode [14]. Van Buren et al. identified IDH in 21.3% of dialysis sessions, with 8% of patients exhibiting persistent IDH [15]. Collectively, these findings suggest that IDH affects approximately one-fifth to one-third of the hemodialysis population, with considerable geographic variation.

Several investigators have shown that IDH is associated with a high-risk clinical profile. Tariq et al. found that IDH occurred exclusively in hypertensive patients and was significantly more prevalent among those with diabetes mellitus (30.9% vs. 12.4%) [4]. In a large cohort study of 37,094 patients, Assimon et al. reported that higher IDH frequency was associated with older age, diabetes, hypertension, and heart failure [10]. Patrice et al. demonstrated a correlation between IDH, hypertension, and the use of two or more antihypertensive medications [14]. These consistent observations suggest that IDH reflects underlying vascular pathology and suboptimal blood pressure control rather than a transient intradialytic phenomenon.

Dialysis-related and treatment-associated factors also play an important role in the pathogenesis of IDH. Van Buren et al. observed significantly lower ultrafiltration rates in patients with chronic IDH (10.4 vs. 12.2 mL/min), indicating inadequate volume removal [15]. Sherif et al. reported associations between IDH and dialysis duration exceeding one year, resistant hypertension, and the use of specific antihypertensive classes such as angiotensin receptor blockers and alpha-blockers. In contrast, beta-blockers and calcium channel blockers were associated with a lower frequency of IDH [13]. Patrice et al. identified intradialytic blood transfusions and polypharmacy with antihypertensive agents as additional risk factors [14]. Furthermore, Assimon et al. demonstrated a consistent association between IDH and the use of dialyzable antihypertensive drugs, supporting the hypothesis that intradialytic drug clearance and vascular dysregulation contribute to paradoxical blood pressure elevation [10].

Notably, volume overload alone does not fully explain the occurrence of IDH. Kale et al. found no significant differences in pre- or post-dialysis weight, ultrafiltration volume, or bioimpedance parameters between IDH and non-IDH groups. However, patients with IDH exhibited a significantly greater intradialytic rise in systolic blood pressure (20.8 ± 8.3 vs. 5.4 ± 13.7 mmHg) [11]. Additionally, lower serum phosphorus levels observed in the IDH group suggest the involvement of metabolic and endothelial mechanisms.

The strongest evidence supporting the clinical relevance of IDH comes from outcome studies. Kale et al. reported higher non-access-related hospitalization rates at six months (60% vs. 26%) and 12 months (60% vs. 42%), along with increased 12-month mortality (40% vs. 15.5%) among IDH patients [11]. In the cohort studied by Assimon et al., patients experiencing IDH in at least 67% of dialysis sessions had significantly higher risks of all-cause mortality (hazard ratio (HR) 2.57) and cardiovascular mortality (HR 3.68), as well as increased rates of all-cause, cardiovascular, and volume-related hospitalizations [10]. Earlier work by Inrig et al. also demonstrated that each increment in intradialytic systolic blood pressure was independently associated with increased mortality [16].

In summary, evidence from prospective studies, cross-sectional analyses, and large cohort investigations indicates that IDH is a common, multifactorial, and clinically significant condition strongly associated with hospitalization and mortality. IDH should therefore be regarded as a high-risk phenotype rather than a minor intradialytic abnormality. Routine identification of IDH, comprehensive assessment of volume status and dialysis prescription, and optimization of antihypertensive therapy may offer substantial opportunities to improve outcomes in patients receiving maintenance hemodialysis

Limitations

This study has a few limitations. As a single-center investigation with a relatively small sample size, the findings may have limited generalizability to the broader hemodialysis population. Additionally, residual confounding from unmeasured variables, including volume status, dietary sodium intake, and medication adherence, cannot be entirely excluded and may have influenced the observed associations.

Multivariable regression analyses, including multiple logistic regression and Cox proportional hazards modeling with HR estimation, were not performed because of the relatively small sample size and the limited number of outcome events. Inclusion of multiple covariates under these circumstances could have resulted in model overfitting and unstable estimates, thereby reducing the reliability of the results. Therefore, the analysis was restricted to Kaplan-Meier survival estimates, and the findings should be interpreted as exploratory and hypothesis-generating rather than confirmatory.

## Conclusions

IDH was observed in a substantial proportion of patients undergoing maintenance hemodialysis and was associated with older age, higher body mass index, longer dialysis vintage, greater cardiovascular comorbidity, and the use of dialyzable antihypertensive medications. Patients with IDH demonstrated higher ultrafiltration volumes, greater interdialytic weight gain, and increased rates of intradialytic adverse events, hospitalizations, cardiovascular complications, and mortality during follow-up. These findings suggest a significant association between IDH and adverse outcomes in the hemodialysis population. Early recognition of IDH during routine dialysis sessions may facilitate patients who could benefit from closer clinical assessment and optimization of management.
